# Soluble factor–mediated enhancement of cell viability via plasma-induced bystander effects

**DOI:** 10.1093/jrr/rrag059

**Published:** 2026-07-26

**Authors:** Misa Iizawa, Masao Suzuki, Ryo Ono

**Affiliations:** Department of Advanced Energy, The University of Tokyo, 5-1-5 Kashiwanoha, Kashiwa, Chiba 227-8568, Japan; Molecular and Cellular Radiation Biology Group, Department of Charged Particle Therapy Research, QST Hospital, National Institutes for Quantum Science and Technology, 4-9-1 Anagawa, Chiba-shi, Chiba 263-8555, Japan; Department of Advanced Energy, The University of Tokyo, 5-1-5 Kashiwanoha, Kashiwa, Chiba 227-8568, Japan

**Keywords:** bystander effect, helium atmospheric-pressure plasma jet, streamer discharge, hormesis-like response

## Abstract

A previous study has shown that gaseous plasma treatment can induce non-monotonic cellular responses, in which cell viability initially decreases with incubation time after treatment but subsequently increases with longer incubation times. In this study, we investigated whether such behavior can be explained by bystander effects mediated by soluble factors released from plasma-treated cells. To this end, conditioned medium obtained from plasma-treated B16F10 and Colon 26 cells was transferred to untreated cells, and the resulting cellular responses were evaluated. The results demonstrated that soluble factors released from damaged cells significantly increased cell viability in untreated cells in a hormesis-like manner. Furthermore, similar responses were observed for 2 different plasma sources, suggesting that this phenomenon is not specific to a particular plasma source. These findings suggest that soluble factor–mediated bystander effects contribute to plasma-induced cellular responses and provide insight into the non-monotonic behavior observed following plasma treatment.

## INTRODUCTION

The application of gaseous plasma in biology and medicine has actively advanced in recent years [1–3]. Plasma is an ionized gas generated by applying a high voltage to gases such as air. In particular, low-temperature atmospheric-pressure plasma is characterized by non-equilibrium conditions in which electrons are highly energetic, while the bulk gas remains near room temperature. Under these conditions, electrons accelerated by an electric field collide with gas molecules and atoms, resulting in ionization as well as the generation of various excited species and radicals through molecular dissociation. Although plasma exhibits certain similarities to ionizing radiation in terms of reactive species generation, a fundamental difference arises from its limited penetration depth. Unlike radiation, which can penetrate biological tissues and induce damage within cells, plasma primarily interacts with the surfaces of liquids and solids, exerting its effects through chemical species generated at or near the interface. Initially introduced for the sterilization of microorganisms, the applications of plasma have since expanded to include hemostasis, wound healing and cancer therapy. Notably, the biological effects of plasma treatment are widely accepted as being primarily mediated by reactive oxygen and nitrogen species (RONS), which induce oxidative stress and modulate intracellular signaling pathways [1–3].

In this context, cancer cells are typically more susceptible than normal cells to plasma-induced oxidative stress [4]. Thus, one proposed cancer therapy mechanism involves the overexpression of aquaporins in cancer cells. Aquaporins are selective membrane channels that facilitate the transport of small molecules such as hydrogen peroxide [2, 4]; consequently, plasma treatment induces a more pronounced increase in intracellular reactive oxygen species (ROS) levels in cancer cells, leading to selective cancer cell death [3–5]. Accordingly, various modes of cell death have been reported as a consequence of this process, including apoptosis, necrosis and immunogenic cell death [1–3].

Plasma treatment can potentially affect not only the directly treated cells but also the surrounding untreated cells, similar to the bystander effects (BEs) observed in radiation biology [3, 5, 6]. In radiation biology, ionizing radiation is known to induce deoxyribonucleic acid (DNA) double-strand breaks and generate harmful species such as hydroxyl radicals, hydrated electrons and hydrogen atoms through the radiolysis of intracellular water. As a result, the radiation-induced BE (RIBE) occurs, wherein irradiation affects not only irradiated cells but also neighboring non-irradiated cells [7–9]. Reported bystander responses include reduced clonogenic survival, increased sister chromatid exchange, apoptosis and altered gene expression. In this context, an early study by Nagasawa and Little [10] demonstrated that α-particle irradiation of Chinese hamster ovary (CHO) cells resulted in increased sister chromatid exchange in ~30% of the cells, despite nuclear traversal occurring in ≤1% of the cell population.

Two major RIBE pathways have previously been proposed: signaling mediated by soluble factors and intercellular communication mediated by gap junctions. In the former pathway, irradiated cells release soluble signaling molecules, such as interleukin-8 (IL-8), transforming growth factor-β1 (TGF-β1) and tumor necrosis factor-α (TNF-α), which activate stress-response pathways in neighboring non-irradiated cells [7]. In the latter, gap junctions, which are widely present in various cell types, physically connect adjacent cells and thereafter enable direct exchange of signaling molecules between irradiated and non-irradiated cells [7–9].

BEs mediated by soluble factors have also been observed in plasma-treated cells. Specifically, using 4 cancer cell lines (A549, A375, J82 and HT29), Zhang *et al.* [11] demonstrated that the conditioned medium (CM) containing soluble factors released from plasma-treated cells reduced the viability of untreated cells by up to 70%, suggesting that plasma-induced soluble factors can mediate antitumor effects. Furthermore, a CM prepared from plasma-treated HaCaT cells selectively reduced the viability of untreated A375 melanoma cells, with no significant effects on untreated HaCaT cells, suggesting cancer-selective BEs mediated by normal cells [12]. In another study, Bauer *et al.* [13] reported that co-culturing a small fraction (0.1–0.2%) of plasma-treated MKN-45 cells with untreated MKN-45 cells significantly increased apoptosis. This effect was attributed to the plasma-induced inactivation of membrane-bound catalase, leading to the secondary generation of singlet oxygen as a soluble factor and subsequent propagation of oxidative stress to the surrounding untreated cells. Despite these findings, however, plasma-induced BEs have not been investigated in sufficient detail.

In addition to these antitumor bystander responses, plasma treatment has been reported to induce non-monotonic cellular responses, including hormesis-like increases in cell viability at low doses and decreased viability at higher doses [14, 15]. Hormesis is a biological phenomenon wherein low stress levels induce adaptive or beneficial responses, whereas excessive stress proves detrimental. However, Mohades *et al.* [16] reported a cellular response distinct from conventional hormesis: specifically, cell viability decreased at 12 h after plasma treatment, whereas longer incubation periods (24 and 48 h) resulted in cell regrowth. This behavior was interpreted as the delayed proliferation of surviving cells.

We hypothesized that the regrowth observed in [16] was mediated by soluble factors released from damaged cells, similar to those known to mediate BEs, which may exert hormesis-like, growth-promoting effects on neighboring or surviving cells. Notably, RIBE has been reported to induce not only deleterious effects but also beneficial responses, including hormesis and adaptive responses [17–19], suggesting that similar mechanisms may be involved in plasma-treated systems.

Based on this background, we demonstrate that soluble factors released from plasma-treated cells can induce increased cell viability in untreated cells in a hormesis-like manner. We further show that the regrowth observed in Ref. [16] can be explained by this mechanism. Finally, our results indicate that the hormetic dose–response behavior often observed during plasma treatment—wherein cell viability initially increases and subsequently decreases with plasma treatment time—can be at least partially explained by soluble factors released from damaged cells.

## MATERIALS AND METHODS

In this study, 2 cancer cell lines—B16F10 and Colon 26—were employed to examine cell-type dependence. Additionally, 2 plasma sources—a helium atmospheric-pressure plasma jet (He-APPJ) and streamer discharge—were employed to examine plasma-type dependence. He-APPJ is widely employed in plasma medicine, whereas streamer discharge has been extensively used by our group to induce antitumor immunity *in vivo* [20–24].

### He-APPJ

A schematic illustration and a photograph of the employed He-APPJ device are depicted in Fig. 1. Two metal tape electrodes (10 mm wide) were wrapped around a quartz tube (with a 4-mm inner diameter) featuring an inter-electrode distance of 20 mm to form high-voltage and grounded electrodes. Helium was supplied from the top of the quartz tube at a flow rate of 1.0 l/min. Positive high-voltage pulses (with 8-kV peak and 1-μs pulse duration) were applied to the high-voltage electrode at a repetition rate of 1000 pulses/s. A 24-well plate was positioned on a grounded metal plate during the plasma treatment. The gap length, defined as the distance between the end of the quartz tube and surface of the culture medium, was 15 mm. He-APPJ generates various reactive species and is often used for medical applications [25].

**Fig. 1:**
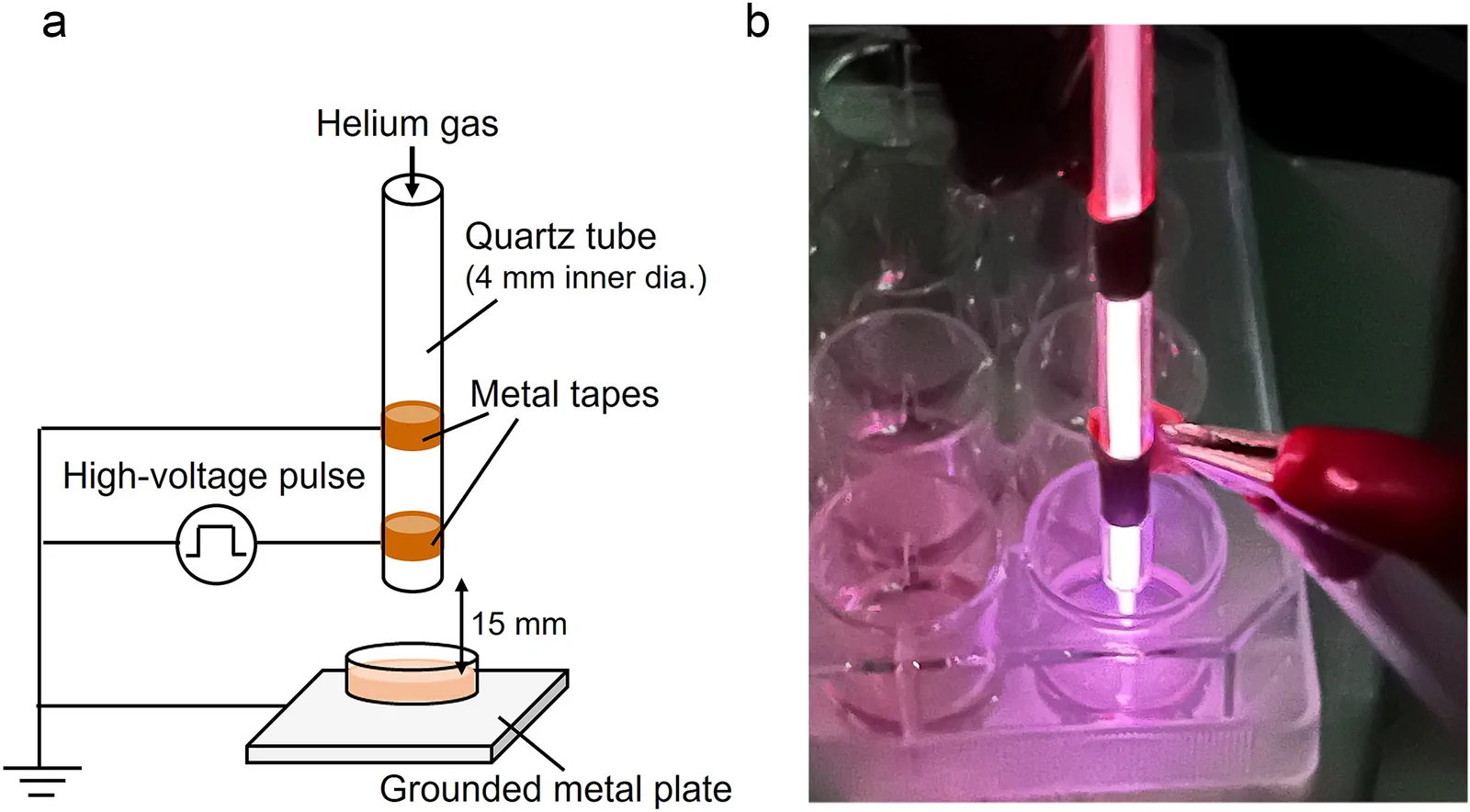
(a) Experimental setup of He-APPJ and (b) its photograph.

### Streamer discharge

Streamer discharge also generates various reactive species [26]. A schematic illustration and photograph of the employed streamer discharge device are shown in Fig. 2. A stainless-steel rod electrode (3 mm in diameter) was inserted into a quartz tube (with a 4-mm inner diameter). A metal tape electrode (5 mm wide) was wrapped around the quartz tube 20 mm from the lower end of the tube. Both the metal tape electrode and metal plate were grounded. Inside the quartz tube, a dielectric barrier discharge was generated between the rod and metal tape electrodes. Under this mechanism, the charged particles produced in this region are transported to the rod–plate electrode gap, enabling stable generation of streamer discharges.

**Fig. 2:**
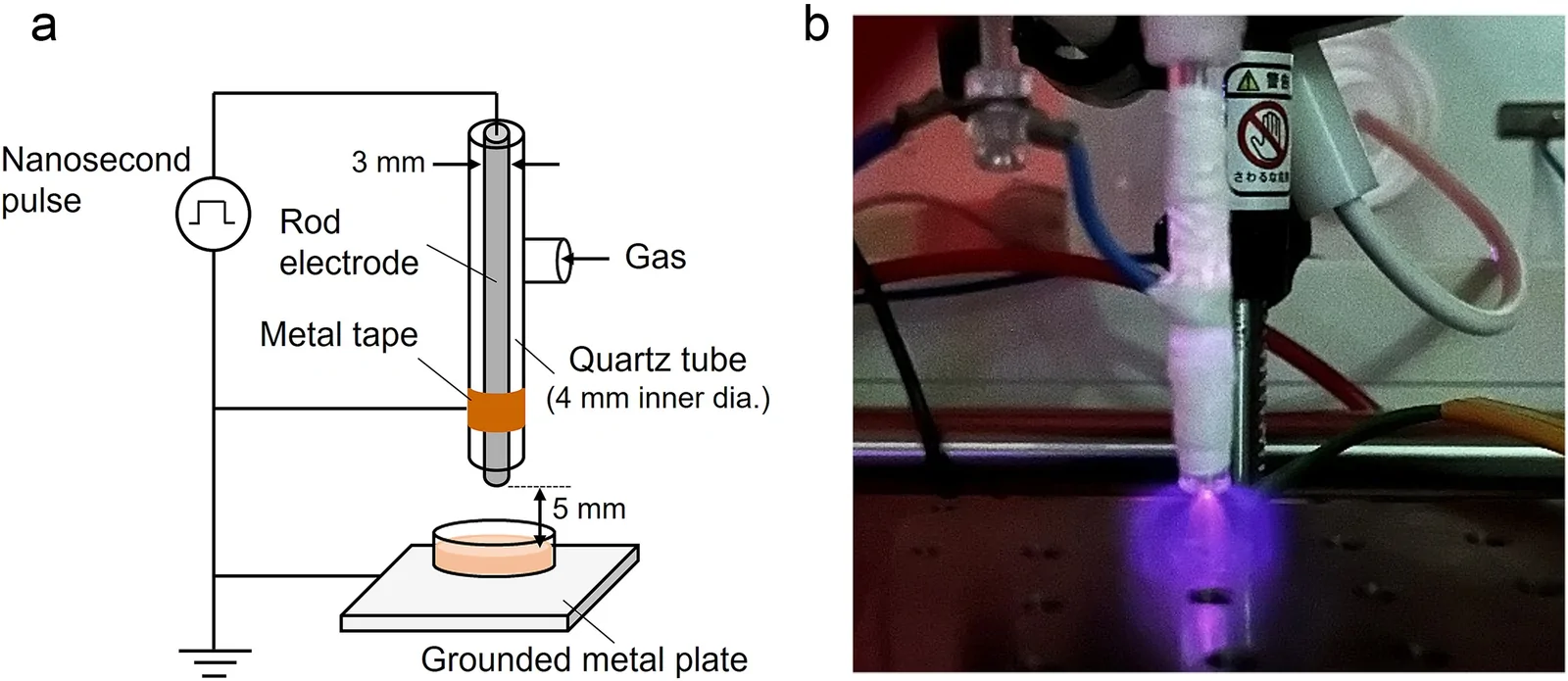
(a) Experimental setup of streamer discharge and (b) its photograph.

The discharge conditions were identical to those adopted in our previous studies [20–24]. Positive high-voltage pulses (with 25-kV peak and 20-ns pulse duration) were applied to the rod electrode at a repetition rate of 100 pulses/s. To enhance ROS generation, humidified oxygen supplied through a bubbling system was introduced from the top of the quartz tube at a flow rate of 0.5 l/min. A 24-well plate was positioned on the grounded metal plate during plasma treatment. The gap length, defined as the distance between the tip of the rod electrode and surface of the culture medium, was 5 mm. The photograph shown in Fig. 2b was captured after removal of the well plate for clear visualization of the discharge.

### Cell culture

The B16F10 cells (RIKEN BioResource Center, Tsukuba, Japan; No. RCB2630) and Colon 26 cells (RIKEN BioResource Center, Tsukuba, Japan; No. RCB2657) were cultured in RPMI medium supplemented with 10% fetal bovine serum and 1% penicillin–streptomycin at 37°C with 5% CO_2_. Subculturing was performed every other day to maintain optimal growth conditions. These 2 cell lines were selected because they have been employed in our previous *in vivo* plasma studies [20–24]. In addition, these cell lines originate from different tumor types (melanoma and colon carcinoma, respectively), enabling evaluation of possible cell-type dependence.

### Soluble factor–mediated bystander experiments

The experimental workflow adopted to evaluate the soluble factor–mediated BEs is shown in Fig. 3 and was designed on the basis of Ref. [11]. Two 24-well plates were prepared—plate A for plasma treatment and plate B for exposure to soluble factors derived from plasma-treated cells. The cells seeded on plate A were defined as treated cells, whereas those seeded on plate B were defined as untreated cells. The initial cell densities were 5 × 10^4^ and 2 × 10^4^ cells/well for the treated and untreated cells, respectively.

**Fig. 3:**
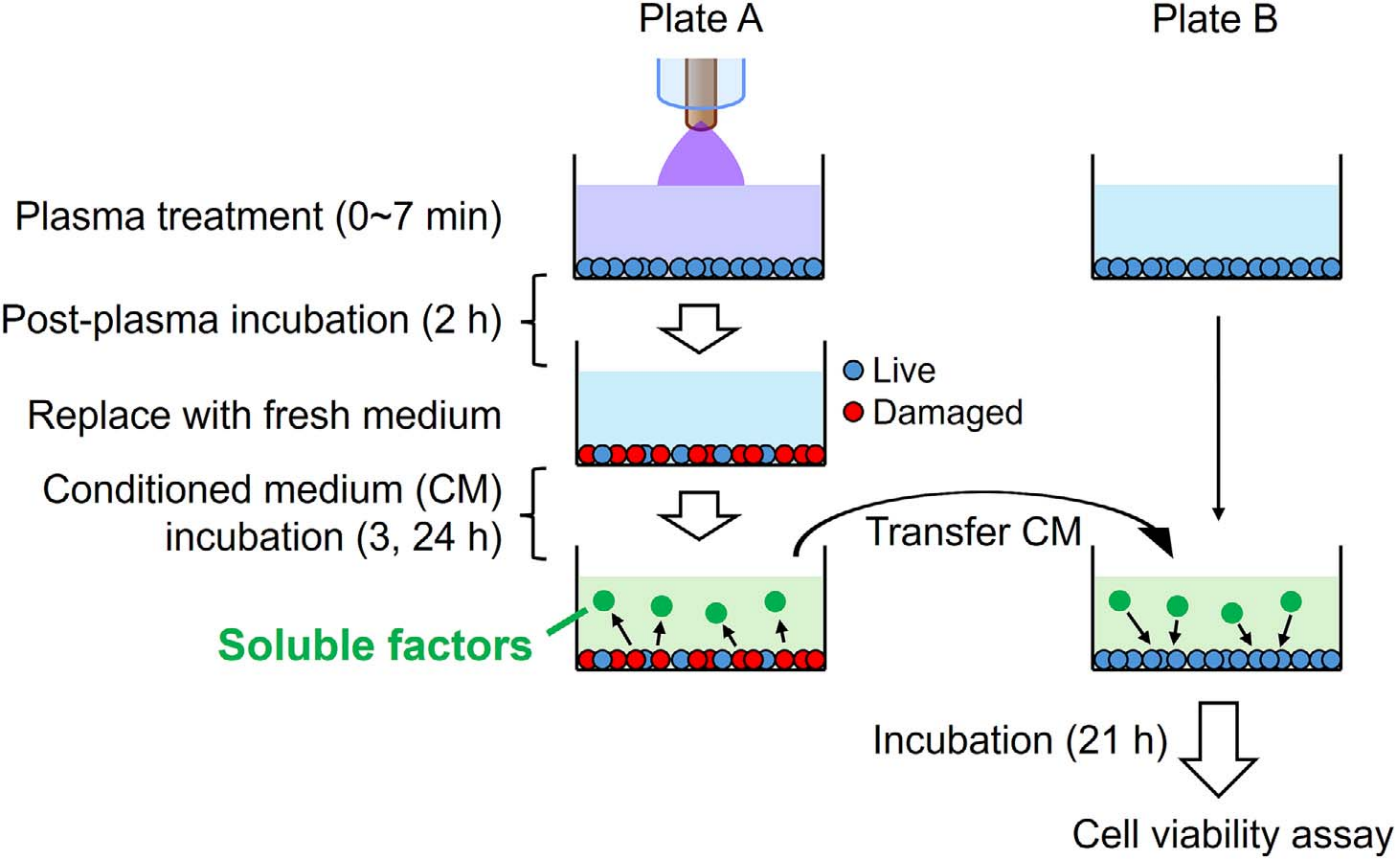
Workflow adopted for evaluating soluble factor-mediated bystander effects.

After the culture medium in plate A was replaced with fresh medium (400 μl per well), each well was exposed to plasma for 0–7 min. During plasma exposure, reactive species were generated in the culture medium and these plasma-generated species are considered to be primarily responsible for inducing cytotoxic effects in the cells. The treated cells were then incubated for 2 h. Subsequently, the medium was replaced with fresh medium to suppress further cytotoxicity induced by the plasma-treated medium, followed by incubation for either 3 or 24 h. During this period, soluble factors released from the treated cells accumulated in the medium, resulting in CM.

After the CM incubation period of 3 or 24 h, the CM was transferred to plate B followed by incubation for an additional 21 h. After incubation, the viability of the CM-treated cells was evaluated using the Cell Counting Kit-8 (CCK-8; Dojindo Laboratories). In this assay, a tetrazolium-based reagent was added to each well, the cells were incubated for 3 h, and the optical absorbance was measured to assess cell viability. Cell viability was defined as the ratio relative to the control.

The number of replicates (*N*) varied depending on the experimental conditions. In each experiment, multiple plasma exposure times were assigned within a single 24-well plate and independent experiments were repeated to increase statistical reliability. For conditions with *n* = 4, data were obtained from a single experiment, whereas larger *N* values (e.g. *n* = 8 or 12) reflect results combined from multiple independent experiments. Additional experiments were performed to ensure reproducibility, resulting in a total sample size ranging from *n* = 4 to 20. For the nutrient control experiment, which was conducted to evaluate the effect of nutrient availability on cell viability independent of plasma treatment, all replicates were obtained within a single experiment.

## RESULTS

### He-APPJ

This section details the results of the He-APPJ experiments. As shown in Fig. 3, a post-plasma incubation time of 2 h was employed in the subsequent bystander experiments; prior to those experiments, an experiment was performed to determine this post-plasma incubation time of 2 h. To this end, the viability of plasma-treated cells was examined as a function of post-plasma incubation time. The results for the B16F10 and Colon 26 cells are shown in Fig. 4. The cells were treated with He-APPJ for 0–7 min and subsequently incubated for 0, 1, 2, 6, or 21 h, after which cell viability was evaluated using the CCK-8 assay.

**Fig. 4:**
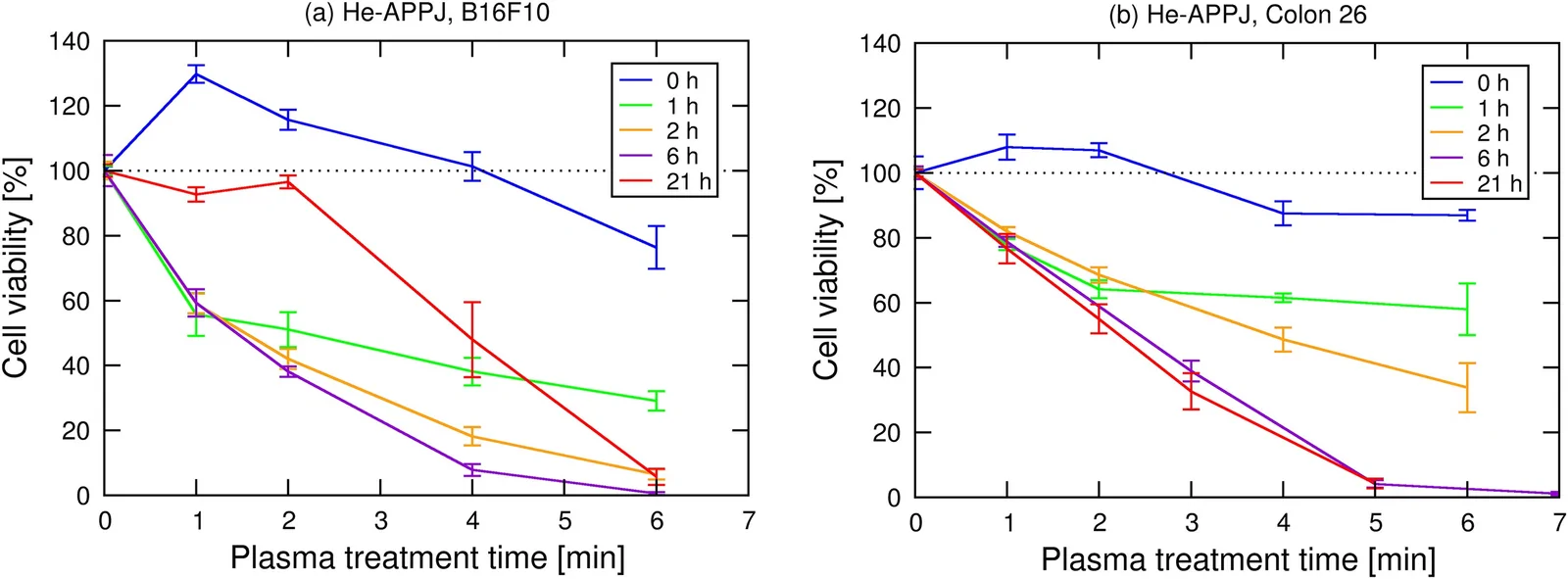
Viability of (a) B16F10 and (b) colon 26 cells on plate A after He-APPJ treatment for 0–7 min with subsequent incubation for 0, 1, 2, 6, or 21 h. The sample sizes ranged from *n* = 4 to 12 for the B16F10 cells and *n* = 4 for the colon 26 cells, depending on the experiment. Error bars represent the standard deviation.

For the B16F10 cells with post-plasma incubation time of 1–6 h and Colon 26 cells with that of 1–21 h, the cell viability monotonically decreased with increasing plasma treatment time and post-plasma incubation time. By contrast, at a post-plasma incubation time of 0 h, an initial increase in cell viability was observed in both cell lines, indicating a hormesis-like response. Based on these observations, and considering that RIBEs are more prominent under low-dose conditions [7] and a post-plasma incubation time of 1 h appears insufficient to capture the expected monotonic decrease in cell viability under longer plasma treatment times, a post-plasma incubation time of 2 h was selected as the appropriate condition for generating CM suitable for bystander experiments. Therefore, this incubation time was adopted for the experiments described in Fig. 3 and subsequent analyses.

Furthermore, as shown in Fig. 4a, B16F10 cells incubated for 21 h exhibited notable regrowth when compared with those incubated for 6 h, consistent with previous findings [16]. This behavior suggests that cell viability at longer incubation times may reflect not only the direct cytotoxic effects of plasma but also secondary responses, potentially involving soluble factors released from damaged cells. Based on this observation, we hypothesized that such soluble factors are responsible for the regrowth. To validate this hypothesis, we next conducted bystander experiments focusing on soluble factor–mediated effects.

The effects of soluble factors were evaluated using the workflow shown in Fig. 3. The results for the B16F10 and Colon 26 cells on plate B are shown in Fig. 5. Soluble factors released over 24 h from plasma-treated B16F10 cells significantly increased cell viability of untreated B16F10 cells by up to ~60% (*t-*test, ^***^P < 0.001), whereas no significant change was observed for the 3-h incubation condition. Similarly, for the Colon 26 cells, soluble factors released over 24 h significantly increased cell viability of untreated cells by up to ~10% (^***^P < 0.001 for plasma treatment times of 2–5 min), whereas no significant effect was observed for the 3-h incubation condition. Although similar trends were observed in both cell lines, the magnitude of response depended on the cell type.

**Fig. 5:**
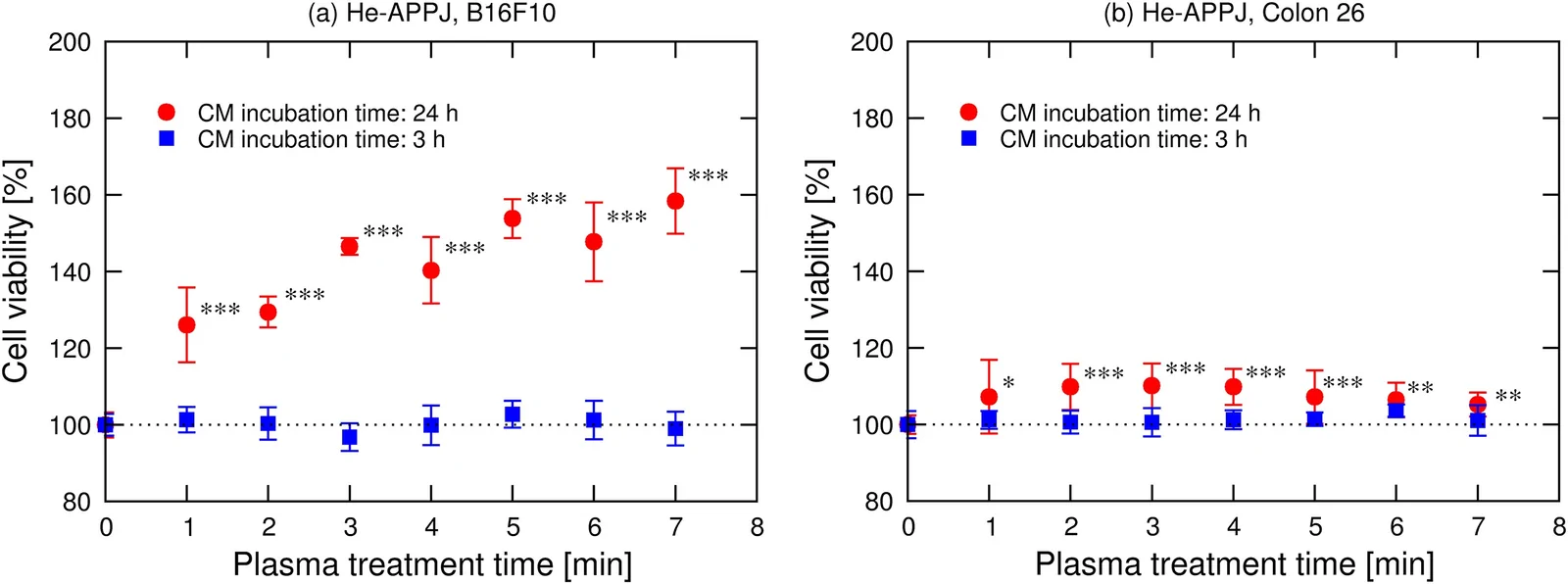
Viability of (a) B16F10 and (b) colon 26 cells on plate B after incubation with CM prepared with CM incubation times of 24 and 3 h (He-APPJ). The sample sizes ranged from *n* = 4 to 20 for both cell types, depending on the experiment. Error bars represent the standard deviation. Statistical significance was evaluated using *t-*test (^*^P < 0.05, ^**^P < 0.01, ^***^P < 0.001).

### Streamer discharge

BEs are often associated with reduced cell viability. However, in this study, increased cell viability was observed in response to soluble factors (Fig. 5). Although several studies have reported proliferative responses associated with RIBE [27–31], such responses have not been clearly demonstrated in the case of plasma-induced BEs. For determining whether the observed phenomenon is specific to the He-APPJ or can also be induced by other plasma sources, identical experiments were conducted using a streamer discharge.

First, the relationship between the post-plasma incubation time and viability of plasma-treated cells was investigated. The results are shown in Fig. 6, demonstrating trends similar to those observed for the He-APPJ (Fig. 4). Based on the trends identified in the He-APPJ experiments, the post-plasma incubation times for the streamer discharge experiments were limited to 2 representative conditions (2 and 21 h), corresponding to substantial cell death and subsequent regrowth, respectively. This approach enabled efficient validation of whether similar behaviors could be reproduced with a different plasma source. Although the overall trends were similar, the extent of regrowth was less pronounced in the streamer discharge experiments than in the He-APPJ experiments.

**Fig. 6:**
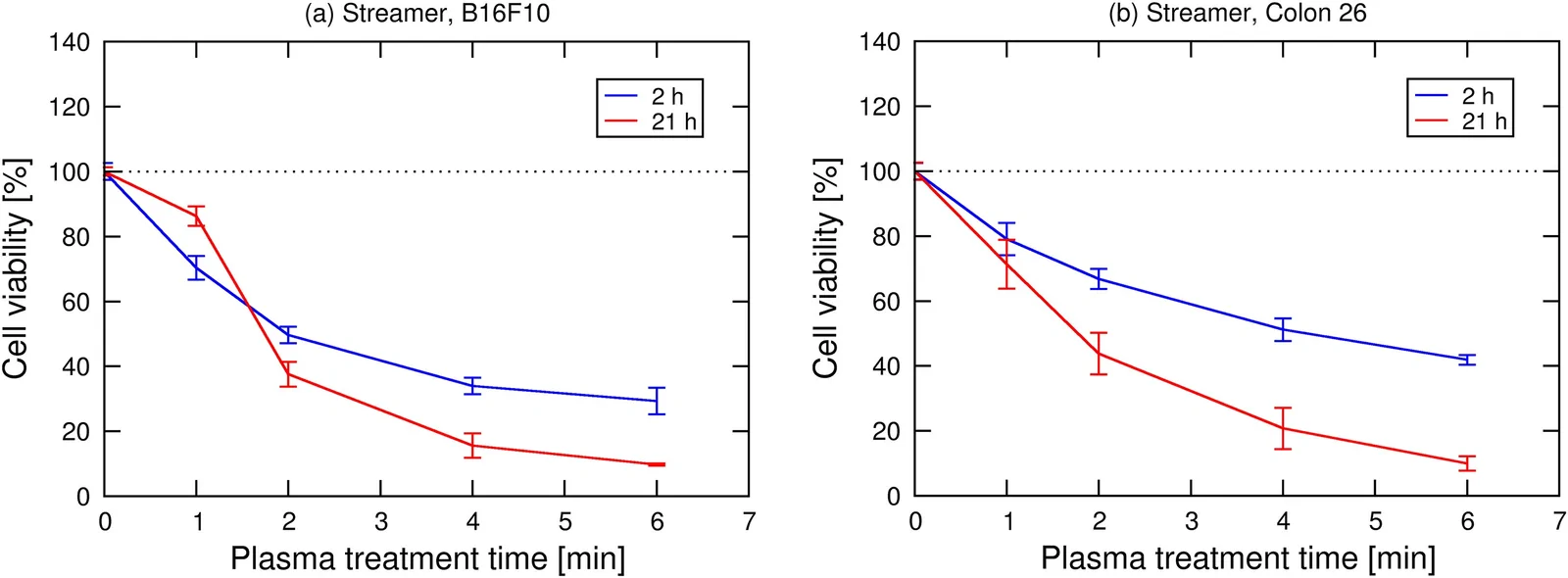
Viability of (a) B16F10 and (b) colon 26 cells on plate A after streamer discharge treatment for 0–6 min with subsequent incubation for 2 or 21 h. the sample size was *n* = 8 for the B16F10 cells and *n* = 12 for the colon 26 cells. Error bars represent the standard deviation.

Accordingly, the post-plasma incubation time for the streamer discharge experiments was fixed at 2 h, which is identical to that adopted in the He-APPJ experiments. Under these conditions, the viability of the plasma-treated cells was comparable between the 2 different discharges (Figs 4 and 6), allowing the effects of the discharge type on soluble factor-mediated cellular responses to be evaluated under similar levels of biological impact. Additionally, the regrowth of cells with longer post-plasma incubation times, as observed in the He-APPJ experiments, was evident in the streamer discharge experiments. As shown in Fig. 6a, for a plasma treatment time of 1 min, the cell viability after 21 h of post-plasma incubation time was higher than that after 2 h of incubation.

The effects of the soluble factors were subsequently examined following the workflow illustrated in Fig. 3; the corresponding results are shown in Fig. 7. Soluble factors released over 24 h from streamer discharge-treated B16F10 cells significantly increased cell viability of untreated B16F10 cells by up to ~60% (^***^P < 0.001), whereas no significant change was observed for the 3-h condition. Similarly, for the Colon 26 cells, soluble factors released over 24 h significantly increased cell viability by up to ~5% (^***^P < 0.001 for plasma treatment times of 1–5 min), whereas no significant effect was observed for the 3-h condition. These trends closely resemble those observed for the He-APPJ. When the treatments were adjusted to achieve comparable viability for the plasma-treated cells, similar soluble factor-mediated responses were yielded for both discharge devices.

**Fig. 7:**
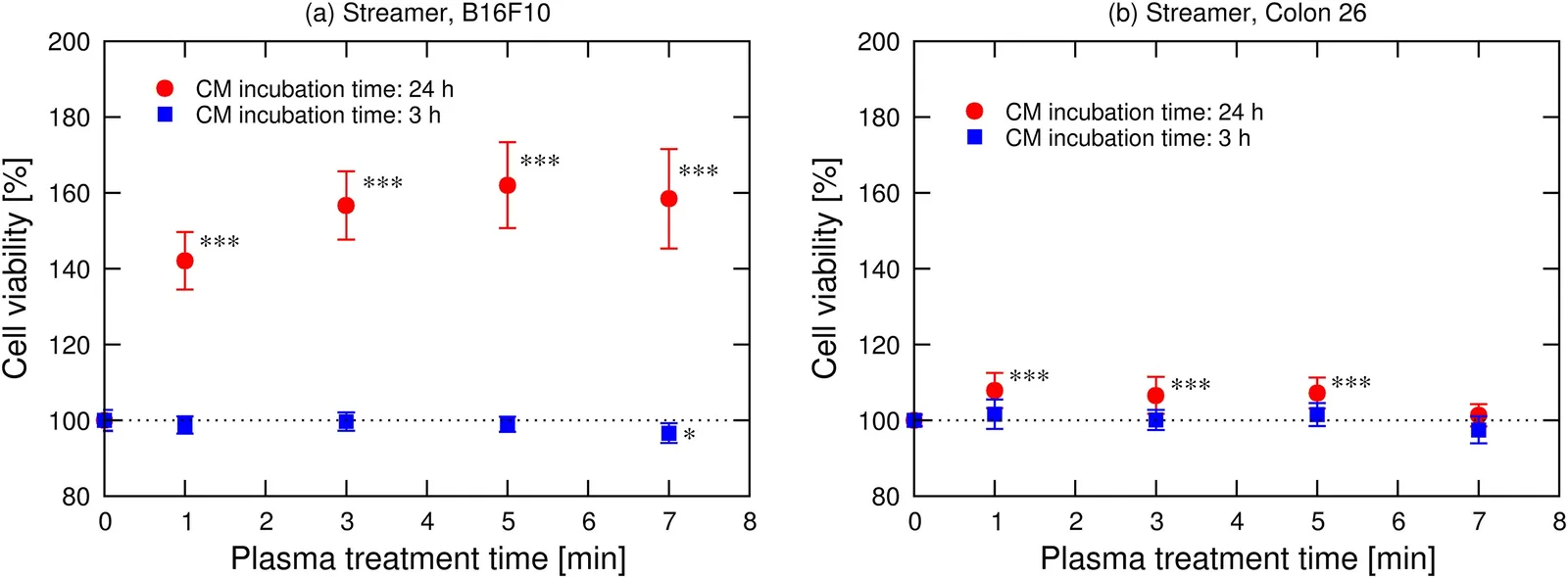
Viability of (a) B16F10 and (b) colon 26 cells on plate B after incubation with CM prepared with CM incubation times of 24 and 3 h (streamer discharge). The sample sizes were *n* = 12 and 8 for CM incubation times of 24 and 3 h, respectively. Error bars represent the standard deviation. Statistical significance was evaluated using *t-*test (^*^P < 0.05, ^**^P < 0.01, ^***^P < 0.001).

### Effect of nutrients consumption in CM

For the experiments detailed in the previous section, the increased viability of untreated cells observed in Figs 5 and 7 was attributed to growth-promoting soluble factor-mediated responses present in the CM. However, an alternative explanation is plausible: as the plasma treatment time increased, the viability of the treated cells decreased, potentially reducing nutrient consumption in the CM. Consequently, the CM with higher residual nutrient levels enhanced the viability of untreated cells.

To investigate this possibility, we assessed the effect of nutrient consumption on cell viability in the absence of plasma by comparing the following 2 conditions:

(i) After 24 h of incubation, the culture medium was replaced with fresh medium, followed by an additional 21 h of incubation before cell viability assessment.
(ii) After 24 h of incubation, the culture medium was replaced with a partially spent medium collected from a separate plate in which the cells had been cultured for 24 h, followed by an additional 21 h of incubation before cell viability assessment.

The results are shown in Fig. 8. Comparable viability was observed overall under both conditions. Notably, a slight but statistically significant increase in viability was observed for B16F10 cells under condition (ii), in which the nutrient level was expected to be lower. This trend is inconsistent with the hypothesis that residual nutrients promote cell viability. Therefore, the enhanced viability of untreated cells observed in Figs 5 and 7 cannot be attributed to differences in nutrient consumption in the CM; instead, it reflects the biological effects of soluble factors released from the plasma-treated cells.

**Fig. 8:**
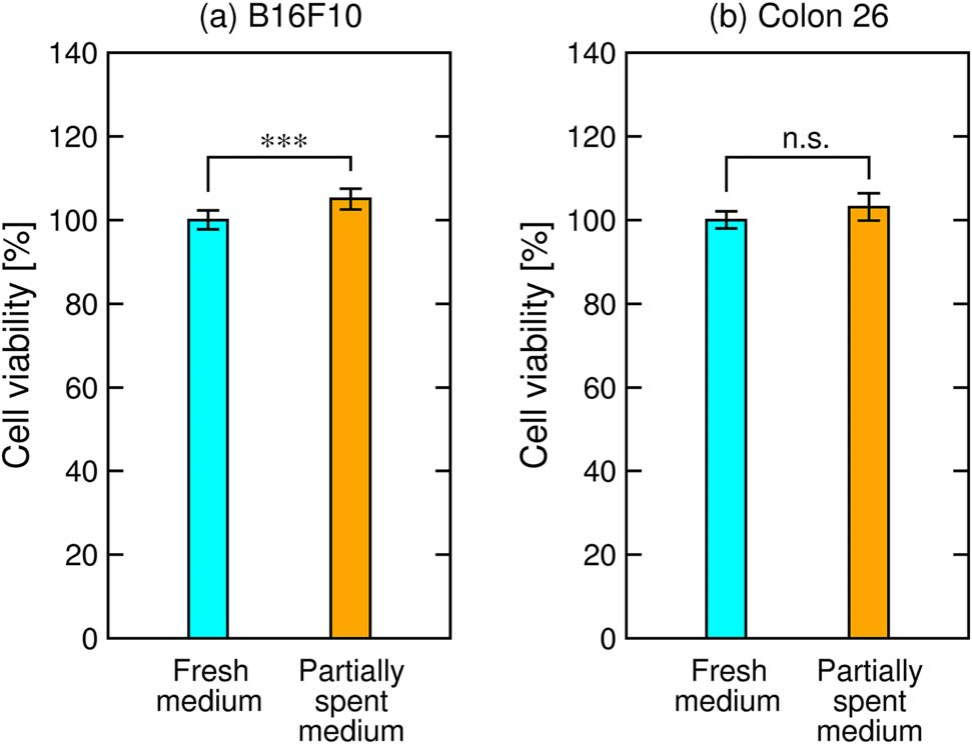
Comparison of cell viability in fresh vs. partially spent medium for (a) B16F10 (*n* = 10) and (b) colon 26 (*n* = 6) cells. Error bars represent the standard deviation. Statistical significance was evaluated using *t-*test (^*^P < 0.05, ^**^P < 0.01, ^***^P < 0.001).

## DISCUSSION

In this study, the CM derived from plasma-treated cells increased the viability of untreated cells (Figs 5 and 7). This observation contrasts with that of Zhang *et al.* [11, 12], who demonstrated substantial reductions in cell viability (by up to 70%) when CM prepared using He-APPJ was applied to various cancer cell lines (A549, A375, J82 and HT29), and a decrease of up to 16% when CM from plasma-treated HaCaT cells was transferred to untreated A375 cells. In those studies, the soluble factors present in the CM were interpreted to induce oxidative stress in untreated cells, resulting in growth inhibition and reduced cell viability. Notably, this effect decreased with shorter plasma treatment times.

One plausible explanation for the discrepancy between these previous studies [11, 12] and our results are that the concentrations of soluble factors in the CM herein did not exceed the threshold required to induce cytotoxic effects in untreated cells. Below this threshold, the CM may act as a low-level stimulus, leading to increased cell viability in a hormesis-like manner. Zhang *et al.* employed a He-APPJ operated with an 8 kV peak-to-peak and 10-kHz sinusoidal waveform—conditions which differed significantly from those adopted in our study. In addition, differences in cell lines and smaller medium volumes (0.3 ml) may have resulted in stronger plasma-induced stimulation in the previous experiments.

Proliferative responses in untreated cells have also been reported after RIBE. Iyer *et al.* [27] suggested that the transfer of supernatant from HFL1 cells irradiated with low-dose α particles (0–1 cGy) to non-irradiated HFL1 cells significantly promotes cell proliferation, with cell numbers increasing to ~135 and 150% of controls on days 2 and 3, respectively. This effect was attributed to the increased concentration of TGF-β1 in the supernatant. Similarly, Gerashchenko *et al.* [28] reported a 17% increase in proliferation of non-irradiated WB-F344 cells following co-culturing with γ-ray-irradiated cells over a dose range of 0.5–20 Gy. Subsequent studies have shown that this proliferative response requires direct cell-to-cell contact via gap junctions [29] and depends on the fraction of irradiated cells [30]. Han *et al.* [31] further reported dose-independent proliferation of non-irradiated CHO cells co-cultured with α-particle-irradiated cells, accompanied by increased DNA damage and they identified NO and TGF-β1 as contributing factors. Collectively, these studies highlight the dual nature of the BE, which can exert either deleterious or beneficial effects depending on the stimulus magnitude and experimental conditions.

In our study, the effects of soluble factors were strongly dependent on CM incubation time. As shown in Figs 5 and 7, little effect was observed at the incubation time of 3 h, whereas a pronounced increase in cell viability was observed after 24 h. These results indicate that soluble factors were predominantly released during later time periods following plasma treatment, specifically after 3 h. Previous studies have reported that plasma-treated cells can generate reactive species such as hydrogen peroxide [32], which may contribute to the observed cellular responses. In addition, secondary reactive species have been suggested to play a role in propagating oxidative stress between cells, as demonstrated by the plasma-induced inactivation of membrane-bound catalase and subsequent generation of singlet oxygen [13]. These findings suggest that reactive species produced by plasma-treated cells may also contribute to the observed bystander-like responses. Although the specific molecular identity, concentrations and temporal profiles of these soluble factors remain to be elucidated, the present results clearly demonstrate their functional role in mediating increased cell viability.

Thus, the present experiments predominantly captured the effects of later-phase soluble factors. On the other hand, because CM was collected only after incubation periods of at least 3 h, the possible contribution of short-lived soluble factors could not be evaluated under the present experimental conditions [7]. Further investigation of early-phase signaling mechanisms and short-lived reactive species will therefore be an important subject for future work.

The results obtained for the post-plasma incubation times of 0 and 21 h in Fig. 4a appear similar, as cell viability did not markedly decrease or even increase with short plasma treatment times. This behavior is often attributed to a conventional hormesis-like response under low-dose plasma stimulation [33, 34]. However, our results suggest that the mechanisms responsible for these responses differ between the 2 conditions. The increase in cell viability observed for the 0-h condition can be interpreted as a conventional hormesis-like response directly induced by plasma exposure. However, when the post-plasma incubation time was increased to 1 h, cell viability decreased even under low-dose plasma stimulation, indicating that the initial hormesis-like effect was no longer evident. With further prolongation of the post-plasma incubation time, cell viability increased again; after 21 h, viability under low-dose stimulation recovered to levels comparable to those of the control. Thus, the apparent similarity between the 0-h and 21-h conditions cannot be explained solely by plasma-induced hormesis-like responses. This observation suggests the involvement of additional mechanisms beyond direct plasma effects.

To better visualize the temporal evolution of cell viability, the data shown in Fig. 4a are replotted in Fig. 9. For plasma treatment times of 1–4 min, cell viability initially decreased at a post-plasma incubation time of 1 h and subsequently increased after 6 h. This behavior can be interpreted as follows: prolonged incubation induces partial cell death and soluble factors released from damaged cells promote increased cell viability of surrounding cells, resulting in net recovery of cell viability. The absence of regrowth at post-plasma incubation times of ≤6 h is consistent with the results shown in Fig. 5a, with no effect of soluble factors observed at a CM incubation time of 3 h. The absence of regrowth in the Colon 26 cells (Fig. 4b) is also consistent with the observation that soluble factor-mediated regrowth was not prominent in this cell line (Fig. 5b). These findings indicate that, in addition to plasma-derived components, soluble factor-mediated responses contribute to the non-monotonic cellular responses observed after plasma treatment. In plasma-induced cytotoxicity studies, cell viability is often evaluated at delayed time points (e.g. 24 h after plasma treatment) rather than immediately after exposure; therefore, when viability curves are similar to those observed for the 21-h condition (Fig. 4a), the possible contribution of soluble factor-mediated effects should also be considered.

**Fig. 9:**
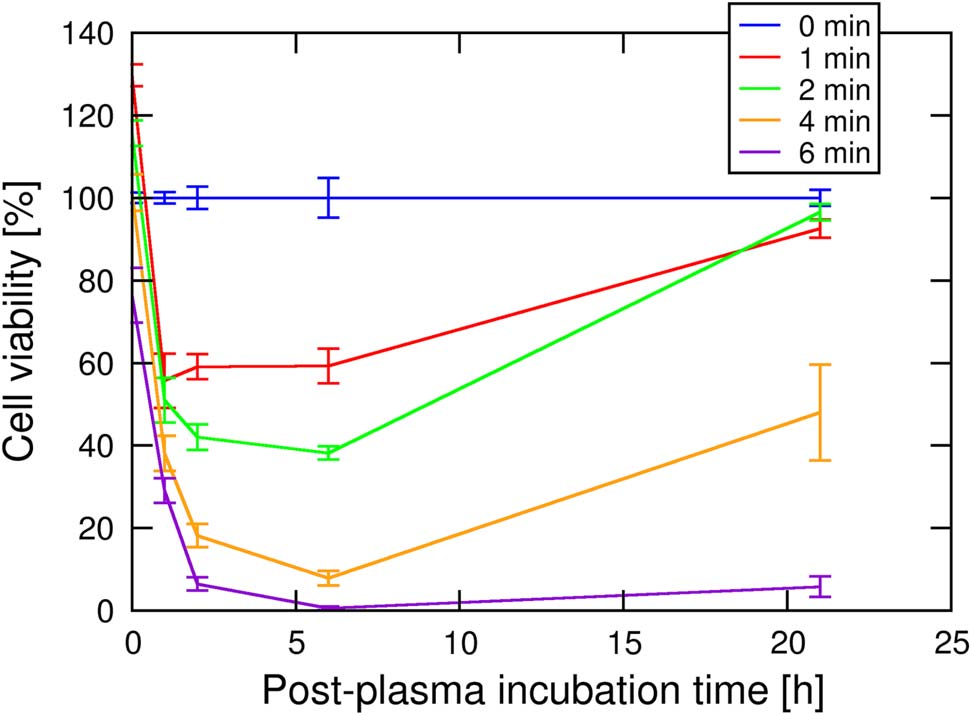
Relationship between post-plasma incubation time and cell viability for B16F10 cells on plate A treated with He-APPJ, as derived from Fig. 4a, with plasma treatment times of 0–6 min. Error bars represent the standard deviation.

A limitation of the present study is that the CCK-8 assay reflects both viable cell number and cellular metabolic activity. Therefore, the observed increases in CCK-8 signal do not distinguish whether the responses originated from enhanced cell proliferation, altered metabolic activity, or both. Additional analyses, such as direct cell counting or clonogenic assays, will be necessary to further clarify the underlying cellular responses.

Plasma-generated long-lived RONS, such as H_2_O_2_, NO_2_^−^ and NO_3_^−^, are widely considered important mediators of biological effects [25]. Similar mechanisms may also contribute to the responses observed in the present study. In particular, Supplementary Fig. S1 shows that the H_2_O_2_ concentration in the culture medium increased with plasma treatment time for both plasma sources. However, the present study did not comprehensively characterize plasma-generated chemical species and the specific soluble factors responsible for the observed responses remain to be elucidated.

## CONCLUSION

The effects of soluble factors were investigated using the He-APPJ and streamer discharge for 2 different cancer cell types. Although the magnitude of the cellular responses depended on the cell type, a significant increase in cell viability was observed in both cell lines at a CM incubation time of 24 h. These results indicate that plasma-treated cells can affect the viability of surrounding untreated cells through soluble factors.

When the plasma treatment conditions were adjusted to achieve comparable viability of the plasma-treated cells, similar soluble factor-mediated responses were yielded for both discharge devices. The possible dual nature of soluble factor-mediated responses, which can exert either deleterious or beneficial effects, was also discussed. In addition, the hormesis-like response often observed after plasma treatment, as part of which cell viability initially increases and subsequently decreases with plasma treatment time, has been discussed in terms of the soluble factors released from damaged cells.

## Supplementary Material

supplementary_rrag059

## PRESENTATION AT A CONFERENCE

Parts of this research were presented at Spring Conferences of the Institute of Electrostatics Japan (IEJ) (2026)
