# Supplementary material for: Soluble factor–mediated enhancement of cell viability via plasma-induced bystander effects
Source: J Radiat Res. 2026 Jul 26;67(5):724–32. doi: 10.1093/jrr/rrag059 (PMC13650710; doi:10.1093/jrr/rrag059)
Supplement: Supplementary file 1 [file supplementary_rrag059.docx]

**Supplementary Data**

Fig. S1. H_2_O_2_ concentration in plasma-treated RPMI medium was measured using a commercial pack test kit (Kyoritsu Chemical-Check Lab., Corp., Japan). For the H_2_O_2_ measurements, transparent RPMI medium was used to avoid color interference with the pack test. Plasma treatment conditions were identical to those described in the main text. Measurements were performed immediately after plasma treatment. Error bars represent the standard deviation obtained from four independent measurements.
